# Numerical Study and Experimental Verification of Biomass Conversion and Potassium Release in a 140 kW Entrained Flow Gasifier

**DOI:** 10.1021/acs.energyfuels.2c03107

**Published:** 2023-01-09

**Authors:** Seyed Morteza Mousavi, Emil Thorin, Florian M. Schmidt, Alexey Sepman, Xue-Song Bai, Hesameddin Fatehi

**Affiliations:** † Department of Energy Sciences, Division of Fluid Mechanics, Lund University, Lund22100, Sweden; ‡ Thermochemical Energy Conversion Laboratory, Department of Applied Physics and Electronics, Umeå University, SE-90187Umeå, Sweden; § RISE AB, Box 726, SE-94128Piteå, Sweden

## Abstract

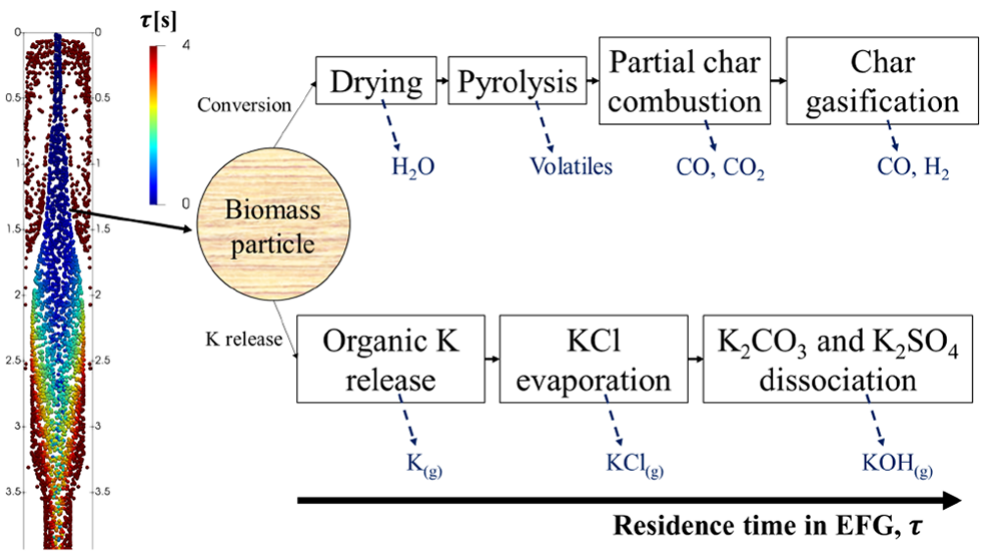

In this study, a Eulerian–Lagrangian model is used to study biomass gasification and release of potassium species in a 140 kW atmospheric entrained flow gasifier (EFG). Experimental measurements of water concentration and temperature inside the reactor, together with the gas composition at the gasifier outlet, are used to validate the model. For the first time, a detailed K-release model is used to predict the concentrations of gas-phase K species inside the gasifier, and the results are compared with experimental measurements from an optical port in the EFG. The prediction errors for atomic potassium (K), potassium chloride (KCl), potassium hydroxide (KOH), and total potassium are 1.4%, 9.8%, 5.5%, and 5.7%, respectively, which are within the uncertainty limits of the measurements. The numerical model is used to identify and study the main phenomena that occur in different zones of the gasifier. Five zones are identified in which drying, pyrolysis, combustion, recirculation, and gasification are active. The model was then used to study the transformation and release of different K species from biomass particles. It was found that, for the forest residue fuel that was used in the present study, the organic part of K is released at the shortest residence time, followed by the release of inorganic K at higher residence times. The release of inorganic salts starts by evaporation of KCl and continues by dissociation of K_2_CO_3_ and K_2_SO_4_, which forms gas-phase KOH. The major fraction of K is released around the combustion zone (around 0.7–1.3 m downstream of the inlet) due to the high H_2_O concentration and temperature. These conditions lead to rapid dissociation of K_2_CO_3_ and K_2_SO_4_, which increases the total K concentration from 336 to 510 ppm in the combustion zone. The dissociation of the inorganic salts and KOH formation continues in the gasification zone at a lower rate; hence, the total K concentration slowly increases from 510 ppm at 1.3 m to 561 ppm at the outlet.

## 1 Introduction

Biomass is a renewable and CO_2_-neutral source of energy that is available worldwide. It is currently the fourth largest source of energy in the world after oil, natural gas, and coal^1^ and is considered a promising replacement for fossil fuels in the future. The concerns about global warming and limited sources of fossil fuels have led to an increased interest in renewable energies over the past few decades. Especially in Europe, the objective is to achieve a 32% renewable target by 2030,^2^ where biomass continues to be the main source of renewable energy with a share of around 60%.^3^ This highlights the importance of biomass as an energy source and the need to further improve the applications and efficiency of biomass conversion devices.

There are various types of devices and technologies for biomass utilization. The biomass particles can be combusted directly in a fixed or fluidized bed reactor, or they can be converted to other types of biofuels through pyrolysis or gasification. The focus of this work is on gasification which is a key solution for biogas generation with high efficiency and low emissions.^4^ In a biomass gasifier, the particles undergo drying, pyrolysis, partial combustion, and char gasification. Char gasification refers to the reaction of char with CO_2_, H_2_O, and H_2_. However, the char reaction with H_2_ is slow and can be neglected in most practical devices.^5^ The produced gas during gasification, which is rich in H_2_ and CO, is called synthesis gas or syngas.^6^ Syngas is a valuable product as it can be cleaned and then used to generate power in engines^7^ or converted to other types of fuels for special applications.^8,9^ One of the main problems in biomass gasifiers is the high content of inorganic elements in biomass such as potassium (K), chlorine (Cl), and sulfur (S) which can cause severe problems, namely, fouling, slagging, corrosion, and agglomeration of bed media.^10^ Further research on different types of biomass gasifiers is still required to improve the quality and yield of the syngas or reduce the negative effects of inorganic elements.

The associated issues with the gasification process, especially those related to the release of inorganic elements and alkali metals, not only are influenced by the fuel type but also are highly dependent on the operating conditions and on the type of gasifier. Biomass gasification can be carried out in fixed bed gasifiers,^11^ bubbling or circulating fluidized bed gasifiers (FBGs),^12−14^ or entrained flow gasifiers (EFGs).^15−17^ The updraft fixed bed gasifiers have a relatively small scale, and they typically lead to the production of high amounts of tars.^11^ The gasification in FBG offers advantages compared to that in a fixed bed as it can be scaled up to medium and large scales, but the temperature should not be too high to prevent agglomeration or too low to avoid too much tar production.^14^ Compared to the other two technologies, EFGs can operate at higher temperatures with small particles, and they can achieve a high carbon conversion rate with low residence time.^15^ Furthermore, in EFGs, the ash can be separated from the products relatively easily, and the high temperature leads to clean and almost tar-free syngas.^6,18^ However, the main drawbacks of EFGs are the problems associated with the release of alkali metals and inorganic elements, soot formation, and other unwanted byproducts such as CO_2_, H_2_O, and CH_4_.^6^ A high temperature and high water vapor concentration, which is a common characteristic of EFGs, promote the release of alkali metals into the gas phase.^19^ This further motivates the studies on the transformation and release of alkali metals in EFGs.

The release of alkali metals and inorganic elements from biomass is commonly studied in lab-scale apparatus on a small batch of biomass particles under pyrolysis, combustion, or gasification conditions.^20,21^ In most experiments, the K content in the solid, as the most abundant alkali metal in biomass, was measured before and after the experiments to calculate the K-release at different operating conditions.^19,22^ It was found that, other than temperature and water vapor concentration, CO_2_ concentration surrounding the particle is important as it prohibits the K-release.^20^ On the other hand, the type of biomass and the composition of the ash-forming elements are also important, since, for instance, Cl and S facilitate while silicon (Si) and aluminum (Al) hinder the K-release.^23^ Other elements such as alkali earth metals, magnesium (Mg), and calcium (Ca) also play an important role as they are favored in reaction with Si, leaving less Si available for reaction with K, and hence promoting the K-release.^24^ With recent advances in optical diagnostics, rapid in situ quantification of gaseous species and characterization of fuel conversion inside reactors up to pilot scale have become possible.^25−28^ Several methods were developed to detect K species in these settings. Laser-induced breakdown spectroscopy (LIBS) can be used to measure the total K concentration in the gas phase.^29,30^ Weng et al.^31^ used UV absorption spectroscopy to measure KOH and KCl in flames. Sorvajärvi et al.^32^ developed collinear photofragmentation and atomic absorption spectroscopy to measure gas-phase K, KCl, and KOH concentrations in a single particle reactor. It was observed that the concentration of atomic K is high during pyrolysis but negligible compared to the KCl and KOH concentrations during the char combustion phase. The KCl release continued during the ash-cooking stage, but at a lower rate compared to the char combustion. More recently, Thorin et al.^33,34^ developed photofragmentation tunable diode laser absorption spectroscopy (PF-TDLAS) for simultaneous measurements of K, KOH, and KCl in both combustion and gasification, the latter usually characterized by optically thick conditions for K. The technique was applied for the detection of the three K species in a 140 kW EFG.^35^ It was shown that the effects of small differences in fuel composition on the potassium release can be resolved by the PF-TDLAS method and that the obtained K species concentrations agreed reasonably well with the results from the thermodynamic equilibrium calculation.

Computational fluid dynamics (CFD) simulation is considered a strong tool to study the performance of biomass conversion systems.^7,36,37^ The most common approach to model the multiphase flow in a gasifier is to use a Eulerian–Lagrangian approach,^4,38−41^ in which the fluid is treated as a continuous phase, and particles are treated as dispersed phases.^42^ Since the number of particles in a real case is very high, a coarse-grain method is commonly used to reduce the computational cost of the model. In the coarse-grain method, the particles with similar properties are grouped together into representative parcels, and the equations are solved only once for each parcel.^43−45^ The CFD models have been carried out to study biomass gasification with a specific focus on syngas production from various sources,^46^ effect of excess air on combustion,^37^ and the effects of key operating parameters on gasification performance.^41,47^ A two-equation soot formation model was also integrated into the CFD model of a gasifier to study the soot emissions.^48^ In most of the above-mentioned numerical models, the model is validated against the temperature or species concentration at the outlet, because experimental data at various heights inside the reactor core are scarce. Therefore, little is known about the flow, reactions, and heat transfer inside the reactor. Furthermore, to the best knowledge of the authors, no detailed model has been used to study the release of potassium species in EFGs.

In this paper, we present a CFD model of an EFG including a K-release submodel which is validated using the experimental measurements of major species at the outlet, and also temperature and H_2_O concentration at two different heights inside the reactor. A detailed K-release model that was recently developed in our group^49^ is integrated into the CFD model to study the release of inorganic elements. The average concentration of different K species across the reactor core at a specific height inside the gasifier is compared with the measurements by Thorin et al.^35^ The model is used to describe the main phenomena happening at different locations inside the reactor. The results from the numerical model are used to explain the experimental observations, and also to explain the main pathways for the transformation and release of the ash-forming elements from the biomass particles during gasification.

## 2 Experimental Measurements

The experiments were conducted in a vertical, 4 m long atmospheric EFG with ceramic lining and an inner diameter of 0.5 m. The reactor was fueled with pulverized forest residues (FR) and oxidizer through a burner at the top. Oxygen-enriched air (54% O_2_) was used as oxidizer and injected at the top at a 30° angle toward the center-line of the reactor.^6^ More details on the facility can be found in the work of Simonsson et al.^50^ The reactor geometry and the burner configuration (Burner number 3) are further described by Ögren et al.^6^ Some of the properties and the elemental composition of the FR fuel used in the experiments are presented in Table 1. The mass fraction of oxygen in the fuel was calculated by mass balance (dry fuel weight minus C, H, N, S, and ash).^35^

**Table 1:** Properties of the Biomass, i.e., the Forest Residue (FR) from Thorin et al.^35^

parameter	value	units
LHV	19.37	[MJ/kg dry]
moisture	6.39	[wt %]
ash	1.84	[wt % dry]
C	51.2	[wt % dry]
H	6.0	[wt % dry]
O	40.0	[wt % dry]
N	0.7	[wt % dry]
K	2500	[mg/kg dry]
Cl	260	[mg/kg dry]
S	480	[mg/kg dry]
Si	2100	[mg/kg dry]
Mg	710	[mg/kg dry]
Ca	3500	[mg/kg dry]

**Figure 1:**
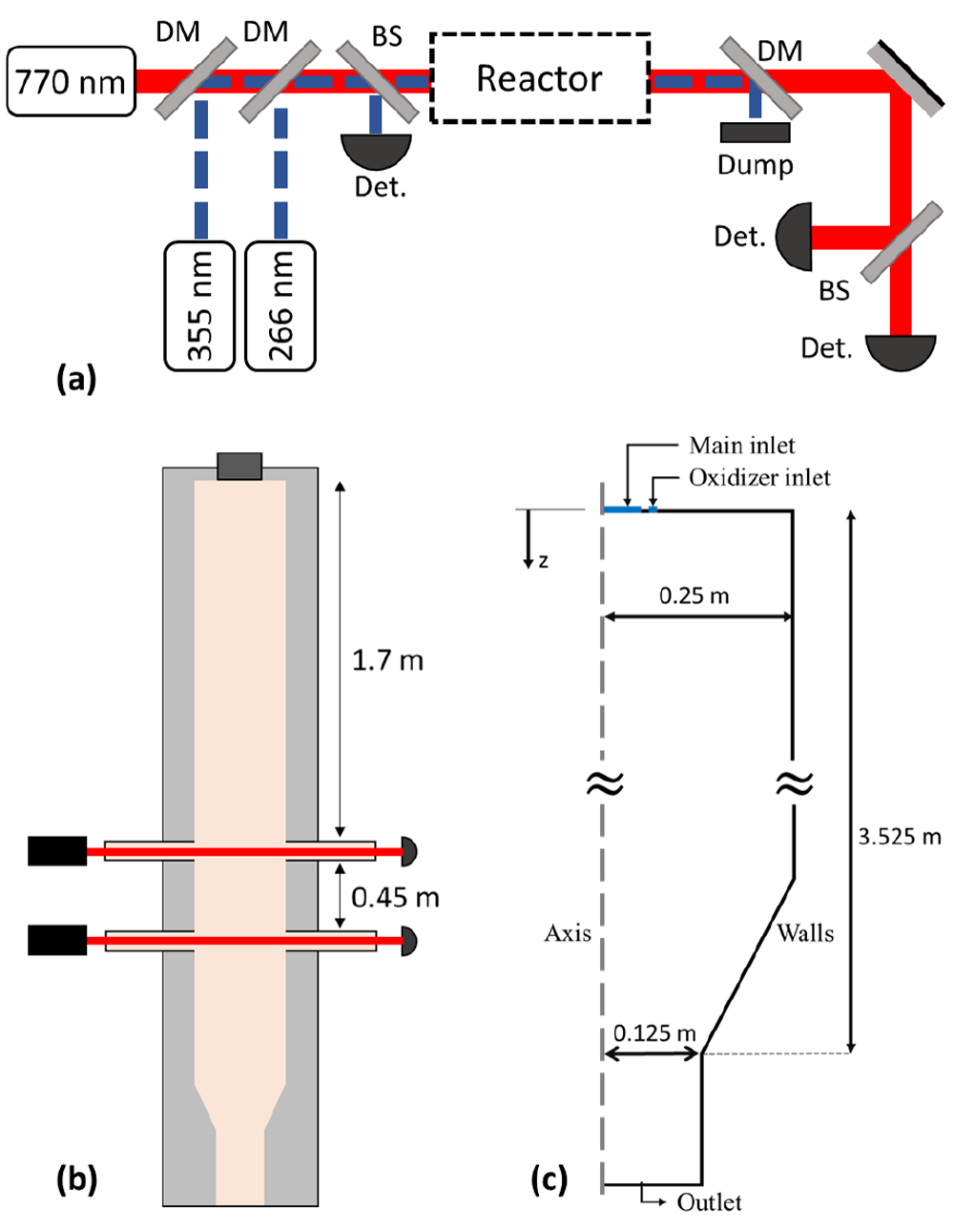
Schematic of the (a) PF-TDLAS system, (b) EFG, and (c) 2D axisymmetric computational domain. DM, dichroic mirror; BS, beam splitter; Det., detector; Dump, beam dump.

Prior to the gasification experiments, the reactor was first preheated by oil combustion until the inner ceramic lining of the reactor reached 1200 K and then by biomass combustion up to a temperature of 1250 K.^35^ In situ laser diagnostics was conducted in the reactor core through optical access ports located 1.7 and 2.15 m downstream of the fuel inlet. Gas temperature and H_2_O concentration were measured at both ports using TDLAS systems operating at 1.4 m,^51,52^ and K species concentrations were measured at the upper port using a PF-TDLAS system.^33−35^ The PF-TDLAS system (Figure 1a) consisted of a tunable diode laser emitting around 770 nm (probe laser) and ns-pulsed Nd:YAG lasers at 266 and 355 nm (pump lasers). The probe laser wavelength was scanned across the atomic K absorption line at 769.9 nm, and the atomic K concentration was obtained by fitting a Voigt profile to the measured absorbance. The UV pump lasers were used to momentarily dissociate KOH and KCl, with subsequent detection of the atomic K fragments with the probe laser. The KOH and KCl concentrations were obtained from the increase in K absorbance due to the fragments.

At the reactor outlet, the concentrations of H_2_O, CO_2_, CO, N_2_, H_2_, and CH_4_ were measured using extractive Fourier transform infrared (FTIR) spectroscopy and micro-gas-chromatography (μGC). Schematics of the EFG and the 2D axisymmetric computational domain used in this study are presented in Figure 1b,c, respectively. The boundary conditions were determined under the operating conditions in the experiments, which are presented in Section 3.5.

## 3 Numerical Model

A Eulerian–Lagrangian approach is used to study the gasification in the reactor. In this method, the solid biomass particles are tracked in a Lagrangian frame of reference, and the fluid phase is described in a Eulerian framework. The particles are modeled using the thermally thin assumption, meaning that the temperature is uniform inside the particle. Because of the axial symmetry in the experimental reactor, a 2D-axisymmetric CFD simulation is carried out using the Reynolds averaged Navier–Stokes (RANS) method.

The governing equations for the continuous fluid phase and the discrete solid particles are presented next. This is followed by the details of the submodels related to particle conversion, gas-phase reactions, and the K-release model. Finally, the boundary conditions and model constants are presented at the end of this section.

### 3.1 Fluid-Phase Governing Equations

The continuity, momentum, species transport, and energy equations for the fluid phase are
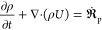
1
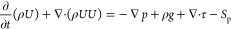
2
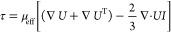
3
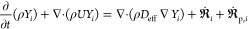
4and
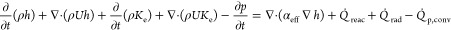
5

In the above equations, ρ, *U*, *p*, and *h* are density, velocity vector, pressure, and the specific enthalpy of the fluid, respectively.  is the gas production rate from the discrete particles. In the momentum equation (eq 2), τ is the total stress tensor, *g* is the gravitational acceleration, and *S*_p_ is the momentum source term from the particles. *Y*_*i*_ is the mass fraction of the *i*th species in the gas mixture, and  and  are the production rate of the same species due to gas-phase reactions and particle conversion, respectively. In the energy equation (eq 5), *K*_e_ is the kinetic energy per unit mass, and *Q̇*_rad_ is the radiation source term. *Q̇*_conv_ is the convective heat transfer from gas to the solid, and *Q̇*_char,g_ is a portion of the heat generated due to heterogeneous char reactions that are directly transferred to the gas phase. The parameters , , *S*_p_, *Q̇*_char,g_, and *Q̇*_p,conv_ are involved in coupling the gas-phase equations to the solid-phase particles. The source terms from the dispersed particles are presented in the next section, and more information regarding the governing equations in the Eulerian–Lagrangian approach was also reported in an earlier study.^36^

The aforementioned quantities are ensemble-averaged, and a turbulence model is required to estimate the total stress tensor, τ, effective dynamic viscosity, μ_eff_, effective mass diffusivity, *D*_eff_, and effective thermal diffusivity, α_eff_. The two-equation κ–ϵ turbulence model is used in this study. The P1 radiation model is used to calculate the radiation source term for both the gas phase and the discrete particles.^53^ A second-order scheme is used for all spatial discretizations, and a first-order implicit scheme is used for time discretization. Grid independence analysis is carried out to make sure that the final results using the mentioned discretization schemes are not dependent on the grid size.

### 3.2 Dispersed Particles’ Governing Equations

The solid particles are tracked in a Lagrangian frame, and the coarse grain method (CGM) is used to reduce the computational time. In CGM, the particles with similar properties at the same location are grouped together in parcels, and the governing equations are solved for parcels instead of particles.^40^ The momentum equation for each parcel is based on Newton’s second law of motion where gravity and drag are the forces acting on the parcel
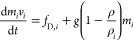
6where *m*_*i*_, ρ_*i*_, and *v*_*i*_ are the mass, density, and velocity of the *i*th parcel, and *f*_D,*i*_ is the drag force acting on the parcel, which is calculated based on the parcel’s velocity relative to the gas-phase velocity (*U* – *v*_*i*_).^36^ The mass and energy balances for the parcels are
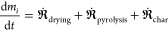
7and
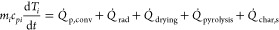
8where the convective heat transfer from surrounding gas to the particles can be calculated by
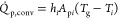
9In the above equations, *c*_p*i*_ and *T*_*i*_ are the specific heat capacity and temperature of the parcel, respectively. *h*_*i*_ is the convective heat transfer coefficient of the parcel which is calculated using the Ranz–Marshall correlation,^54^ and *A*_p*i*_ is the parcel surface area. *Q̇*_rad_ is the net radiative heat transfer to the parcel and is calculated based on the same radiation model as in the fluid phase. In the mass balance equation, , , and  are the mass losses due to drying, pyrolysis, and char conversion, respectively. Similarly, *Q̇*_drying_, *Q̇*_pyrolysis_, and *Q̇*_char,s_ are the heat sources due to the various stages of particle conversion. The particle conversion and the submodels for drying, pyrolysis, and char oxidation/gasification, which are used to calculate the above-mentioned source terms, are explained in the following.

### 3.3 Particle Conversion

The raw biomass particle is a porous solid that initially contains some amount of moisture. By injecting the particle into the reactor, the particle temperature increases which leads to drying followed by pyrolysis. During drying, the particle moisture evaporates, and during pyrolysis, the particle volatile content is released in the form of gas and tar (volatiles that are in condensed form at room temperature) species. After pyrolysis, the solid residue consists mainly of char and ash. Depending on the atmosphere surrounding the solid residue, the char is either combusted with O_2_ or gasified through reactions with H_2_O and CO_2_. The release of ash-forming elements such as K, Cl, and S can occur at every stage of particle conversion, or even after char conversion (ash cooking stage).

In the present model, the initial mass fractions of liquids, *y*_L_, volatiles, *y*_V_, and solids, *y*_S_, are model inputs which can be estimated based on the biomass characteristics and the operating conditions. Some of the properties of the forest residue (FR) that is investigated in the present study are presented in Table 1. *y*_L_ can be calculated based on the moisture content of the fuel, and we have used a detailed single-particle model^55^ to estimate the *y*_V_ and *y*_S_ corresponding to pyrolysis in a condition similar to the top of EFG where the particles are injected. The mass fractions of liquids, volatiles, and solids for the FR in the present study are estimated to be *y*_L_ = 0.06, *y*_V_ = 0.73, and *y*_S_ = 0.21. The liquids, volatiles, and solids are converted and released to the gas phase based on the drying, pyrolysis, char conversion, and alkali release submodels that are explained in the following.

#### 3.3.1 Drying

An equilibrium model is used to model particle drying, which is based on the assumption of equilibrium between water in liquid and gas phases.^56^ In this model, the drying rate is estimated by
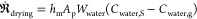
10where *h*_m_ is the mass convection coefficient, *A*_p_ is the particle surface area, and *W*_water_ is the molecular weight of water. *C*_water,S_ and *C*_water,g_ are the water vapor concentrations at the particle surface and in the gas phase surrounding the particle, respectively. The *C*_water,S_ is calculated by the saturation vapor pressure of the water at the particle surface temperature. The heat of drying can be calculated based on the drying rate by , where *Δh*_vap,water_ is the latent heat of water evaporation.

#### 3.3.2 Pyrolysis

The pyrolysis is modeled using a single-step global reaction where the volatile content of biomass is converted to various gaseous products. Six volatile species are considered here that are H_2_, H_2_O, CO, CO_2_, CH_4_, and C_2_H_6_, as representatives of the pyrolysis products and tar decomposition process. The mass fractions of the volatile species that are presented in Table 2 are estimated using the elemental composition and LHV of the fuel by a method explained earlier.^57^ The pyrolysis is modeled using a first-order Arrhenius reaction rate that is expressed by
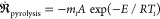
11Various kinetic rates are reported in the literature,^58^ but here, the rates of gas and tar production from Thurner and Mann,^59^ i.e., *A* = 1.73 × 10^6^ s^–1^ and *E* = 106.5 *kJ*/mol, are used to model the pyrolysis. A wide range of data is reported in the literature for the heat of pyrolysis, *Q̇*_pyrolysis_, due to the different biomass types and experiment conditions.^60^ However, the magnitude of pyrolysis heat is small compared to the heat of char conversion and is neglected in the current study.

**Table 2:** Mass Fractions of Species in the Volatile Mixture

species	H_2_	H_2_O	CO	CO_2_	CH_4_	C_2_H_6_
*Y*_*j*_	0.05	0.06	0.58	0.22	0.08	0.01

#### 3.3.3 Oxidation and Gasification

After pyrolysis, the solid particle consists of char and ash, and the char conversion is governed by heterogeneous oxidation with O_2_ or gasification with CO_2_ and H_2_O. The heterogeneous char reactions are very complex as they depend on many variables which affect the kinetics such as temperature, partial pressure of the gasifying agent and the product gases, and composition of inorganic matter. The heterogeneous reactions are also influenced by the morphological structure, size, and porosity of the particles which affects the diffusion rate of the gasifying agent.^5,14^ The char conversion with any of the gasifying agents takes place in several steps, but the majority of the kinetic analyses simply consider a single-step global reaction for char gasification and oxidation.^5^ Also in this study, three global reactions, R1–R3, as presented in Table 3 are used to model the char oxidation and gasification. The parameter Ω_C_ in R1 determines the CO/CO_2_ ratio during char combustion, and it can be calculated based on particle temperature:^61,62^
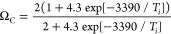
12

**Table 3:** Kinetic Rate Constants for Heterogeneous Oxidation and Gasification Reactions^a^

	reaction	*R*_kin,i_/*m*_C_[1/s]	ref
R1	Ω_C_C + O_2_ → 2(Ω_C_ – 1)CO + (2 – Ω_C_)CO_2_	(7.06 × 10^5^)*P*_O_2__^0.78^ exp(−160 × 10^6^/*RT*)(1 – *X*)	(64)
R2	C + CO_2_ → 2CO	(3.62 × 10^4^)*P*_CO_2__^0.8^*T*^–0.8^ exp(−166 × 10^6^/*RT*)(1 – *X*)^2/3^	(65)
R3	C + H_2_O → H_2_ + CO	(1.773 × 10^3^)*P*_H_2_O_^0.41^ exp(−179 × 10^6^/*RT*)	(66)

a Units in [Pa J kmol m s K].

The rate of the heterogeneous reactions can be limited by both kinetics and mass diffusion of the gasifying agent. The char conversion can happen in three different regimes based on the Thiele modulus, which is the ratio of the overall reaction rate to the internal diffusion rate, or the Thiele modulus squared, which is the internal Damköhler number.^5,14^ The char reactions in regimes I, II, and III are limited by kinetics, pore (internal) diffusion, and external mass transfer to the particle, respectively.^14^ The intrinsic kinetic rate of char conversion has to be measured in regime I, which is for very small particles (smaller than 60 μm).^5^ However, in most practical devices, the conversion regime is between regimes I and III, so both external diffusion and kinetic rates are important.^63^ Hence, a kinetic-diffusion limited rate is used for the char conversion rate,^36,48^ which can be expressed in a general form as
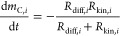
13where *i* is the gasifying agent (O_2_, CO_2_, or H_2_O), *R*_diff,*i*_ is the external diffusion rate, and *R*_kin,*i*_ is the intrinsic kinetic rate for each gasifying agent. The diffusion rate can be calculated by
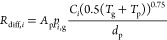
14where *A*_p_ and *d*_p_ are the particle surface area and diameter, respectively. *C*_*i*_ is estimated based on the mass convection coefficient and is equal to 5 × 10^–12^ν_C,*i*_,^36^ assuming the particles are flowing with the same velocity as the fluid. ν_C,*i*_ is the stoichiometric ratio of char to the gasifying agent and is equal to Ω_C_ for char combustion and equal to 1 for gasification reactions. The kinetic rate is in a general form expressed as
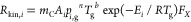
15where *F*_*X*_ defines the dependence of the reaction rate on the char conversion due to changes in the active surface area of char, where the char conversion is quantified using X = (*m*_C,0_ – *m*_C_)/(*m*_C,0_ – *m*_C,∞_).^5^ The variables *m*_C_, *m*_C,0_, and *m*_C,∞_ are the current, initial, and final mass of char, respectively. The kinetic constants of the heterogeneous reactions are presented in Table 3.

#### 3.3.4 Potassium Release

The release of K, Cl, and S from solid biomass is simulated using the K-release model that was developed recently in our group.^49^ The model consists of 12 solid-phase species and 13 reactions, and it can predict the transformation of various types of K, Cl, and S species during biomass conversion. The K-release reactions and their corresponding rates in Arrhenius form are presented in Table 4. The evaporation rates of KCl, K_2_CO_3_, and K_2_SO_4_ are calculated using a mass transfer limited evaporation model.^49^

**Table 4:** Reactions and Rate Constants Used to Model the Transformation and Release of K-, Cl-, S-, and Si-Containing Species^49^^a^

	reaction	rate
PR1	K_inorganic_ → ϕ_1_KCl + ϕ_2_ K_2_SO_4_ + ϕ_3_ K_2_CO_3_	5.13 × 10^10^ exp(−88 × 10^6^/*RT*)
PR2	S^2–^ → S_crystal_	5.13 × 10^10^ exp(−88 × 10^6^/*RT*)
PR3	S_crystal_ → SO_2(g)_	1.25 × 10^11^ exp(−125 × 10^6^/*RT*)
PR4	KCl + R–COOH → R–COOK + HCl_g_	1.25 × 10^11^ exp(−125 × 10^6^/*RT*)
PR5	R–COOK → char-K + CO_2(g)_	1.10 × 10^7^ exp(−112 × 10^6^/*RT*)
PR6	R–COOK → R + CO_2(g)_ + K_g_	4.40 × 10^9^ exp(−153 × 10^6^/*RT*)
PR7	KCl → KCl_g_	evaporation model
PR8	K_2_CO_3_ → 2K_g_ + CO_2(g)_ + 0.5O_2(g)_	evaporation model
PR9	K_2_CO_3_ + H_2_O_g_ → 2KOH_g_ + CO_2(g)_	3.25 × 10^14^ exp(−360 × 10^6^/*RT*)*p*_H_2_O_^*n*^
PR10	K_2_SO_4_ → K_2_SO_4(g)_	evaporation model
PR11	K_2_SO_4_ + H_2_O_g_ → 2KOH_g_ + SO_2(g)_ + 0.5O_2(g)_	5.93 × 10^8^ exp(−261 × 10^6^/*RT*)*p*_H_2_O_^*n*^
PR12	char-K → αK_2_CO_3_ + (1 – α)K_2_SiO_3_	2.00 × 10^7^ exp(−182 × 10^6^/*RT*)*X*_char_
PR13	K_2_SO_4_ + SiO_2_ → K_2_SiO_3_ + SO_2(g)_ + 0.5O_2(g)_	2.00 × 10^6^ exp(−182 × 10^6^/*RT*)*X*_char_

a Units in [kg J kmol m s K].

In this model, potassium is initially in the form of K_inorganic_, organic R–COOK, or stable K_2_SiO_3_. Sulfur is initially in the form of organic S^2–^ or inorganic K_2_SO_4_, and chlorine is in inorganic KCl form. The initial ratio of organic to inorganic K, Cl, and S in the fuel is estimated based on the experimental observations for various fuels.^49^ The parameters ϕ_1_, ϕ_2_, and ϕ_3_ in PR1 and α in PR12 are calculated based on the ash composition of the fuel as explained in Section 3.5.

### 3.4 Gas-Phase Reactions

A set of global gas-phase reactions that are used in previous studies on biomass gasification^36,40,41,67^ is considered here. In addition, another global reaction is added to include C_2_H_6_ combustion with oxygen.^68^ The gas-phase reactions and their rate constants are presented in Table 5. R4 is the reaction of methane with water that only becomes important at very high temperatures. R5 and R6 are the reversible water–gas shift reactions. Reactions R7–R10 are responsible for the combustion of CH_4_, CO, H_2_, and C_2_H_6_ with oxygen, respectively.

**Table 5:** Gas Phase Reactions and Their Kinetic Rates^67,68^^a^

	reaction	rate
R4	CH_4_ + H_2_O → CO + 3H_2_	0.312 exp(−126 × 10^6^/*RT*)[CH_4_][H_2_O]
R5	CO + H_2_O → CO_2_ + H_2_	2.5 × 10^8^ exp(−138 × 10^6^/*RT*)[CO][H_2_O]
R6	CO_2_ + H_2_ → CO + H_2_O	9.43 × 10^9^ exp(−171 × 10^6^/*RT*)[CO_2_][H_2_]
R7	CH_4_ + 2O_2_ → CO_2_ + 2H_2_O	2.1 × 10^11^ exp(−203 × 10^6^/*RT*)[CH_4_]^0.2^[O_2_]^1.3^
R8	CO + 0.5O_2_ → CO_2_	1.0 × 10^10^ exp(−126 × 10^6^/*RT*)[CO][O_2_]^0.5^
R9	H_2_ + 0.5O_2_ → H_2_O	2.2 × 10^9^ exp(−109 × 10^6^/*RT*)[H_2_][O_2_]
R10	C_2_H_6_ + 3.5O_2_ → 2CO_2_ + 3H_2_O	3.5 × 10^7^ exp(−126 × 10^6^/*RT*)[C_2_H_6_]^0.1^[O_2_]^1.65^

a Units are [J kmol m s K].

### 3.5 Boundary Conditions and Model Parameters

The boundary conditions in the simulations are set according to the experimental conditions during the gasification of FR with an air-fuel equivalence ratio (AFR) of 0.5.^52^ Air and fuel are injected through the main inlet, and oxygen is injected through the oxidizer inlet (Figure 1c). The mass flow rate of air and fuel from the main inlet and the mass flow rate of oxygen from the oxidizer inlet are presented in Table 6. The temperature of the fuel, air, and oxygen is 300 K at the inlets, and the outlet pressure is 1 atm. The wall temperature was measured at eight different heights, and here, a third-order polynomial is used to best fit the measured data. A Rosin–Rammler distribution is used to represent the FR particle size at the inlet in the form of *Y*_d_ = exp[−(*d*/*d̅*)^*n*^], where *Y*_d_ is the mass fraction of particles with diameter greater than *d*, *d̅* is a size parameter, and *n* is a distribution width parameter.^69^ The Rosin–Rammler constants that are presented in Table 6 are calculated based on the measurements reported earlier.^35^

**Table 6:** Boundary Conditions and the Model Parameters Used for the K-Release Model

parameter	value
Boundary Conditions	
main inlet *ṁ*_fuel_[kg/h]	27.4
main inlet *ṁ*_air_[kg/h]	16.9
oxidizer inlet *ṁ*_O_2__[kg/h]	14.3
inlet temperature [K]	300
wall temperature, *T*_*z*_ [K]	17*z*^3^ – 110*z*^2^ + 99*z* + 1434
outlet pressure [atm]	1
Rosin–Rammler *d̅* [μm]	398
Rosin–Rammler *n*	1.9
Parameters	
ϕ_1_ (mass-based)	0.14
ϕ_2_ (mass-based)	0.27
ϕ_3_ (mass-based)	0.59
α	1

The parameters used in the potassium release model are also presented in Table 6. ϕ_1_ is calculated based on the assumption that all Cl in fuel forms KCl after particle drying. Around 60% of S is in organic form,^19^ and the rest is in the form of K_2_SO_4_, which is used to calculate the ϕ_2_ value. ϕ_3_ is calculated by the mass balance of total potassium in inorganic form. The FR fuel has low Si content, so all potassium in char-K is assumed to be converted to K_2_CO_3_ through reaction PR12, which leads to α = 1. A detailed discussion on the model parameters, ϕ_1_, ϕ_2_, ϕ_3_, and α, and the choice of model parameters for different types of fuel is explained in more detail in ref (49).

## 4 Results and discussion

### 4.1 Grid Independence Analysis

**Figure 2:**
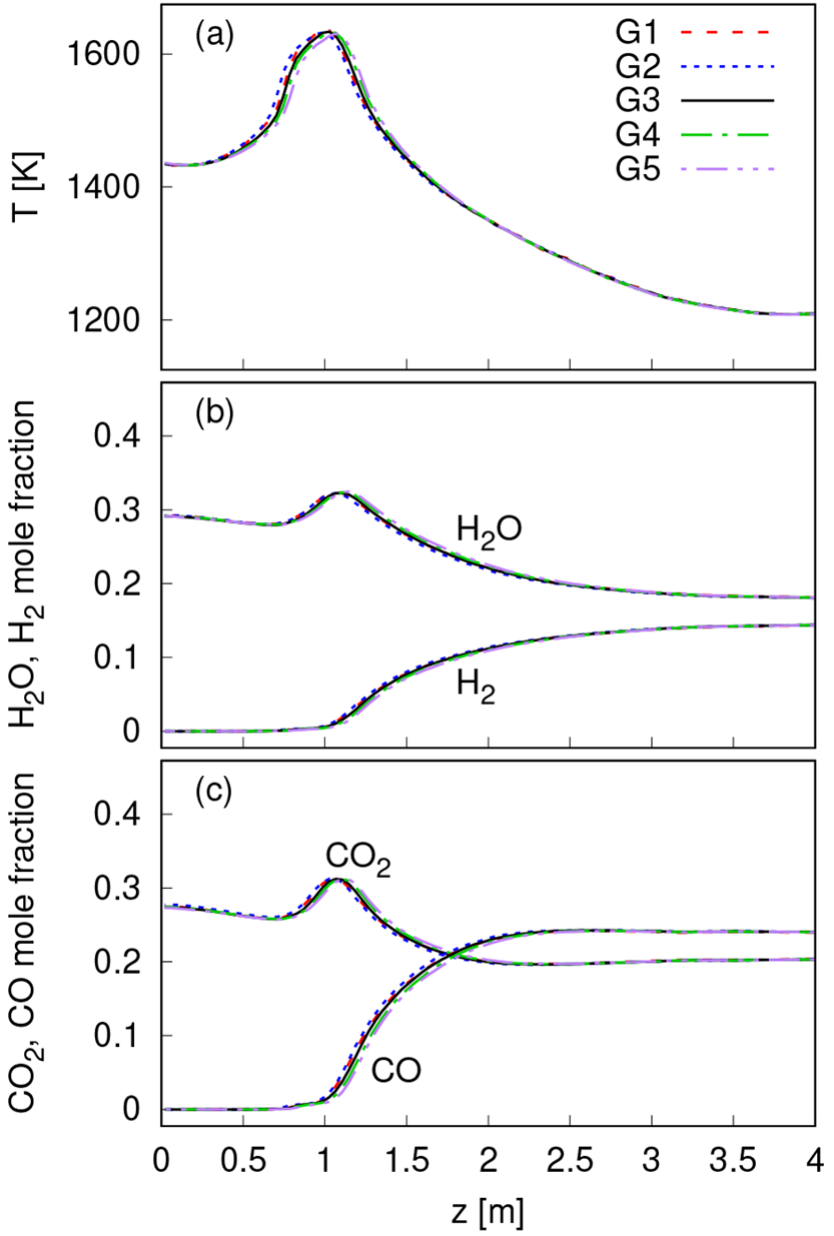
Sensitivity of the results to the grid resolution. Average temperature (a), H_2_O and H_2_ mole fractions (b), and CO_2_ and CO mole fractions (c) along the reactor vertical axis.

The grid independence study is performed using five different grids, named G1–G5, with a different number of cells that are presented in Table 7. Furthermore, the root-mean-square deviation (RMSD) of temperature normalized by maximum temperature and relative to the finest grid case is presented in the same table. The average temperature and the mole fractions of H_2_O, H_2_, CO_2_, and CO along the reactor vertical axis for the five cases are presented in Figure 2. In all cases, 30,000 parcels per second are injected into the domain. It was also observed that the results are not sensitive to the number of injected parcels in the range of 20,000–40,000 parcels per second (the results are not presented here). Case G3 is used in the rest of the paper as it leads to a low error and a considerably lower computational cost compared to G5.

**Table 7:** Five Cases That Are Used for Grid-Independence Analysis^a^

case	G1	G2	G3	G4	G5
grid cells [10^3^]	9.4	21.0	36.0	58.4	84.0
RMSD of temperature [%]	2.1	0.9	0.5	0.25	0

a RMSD is based on case G5.

### 4.2 Temperature and Major Species

**Figure 3:**
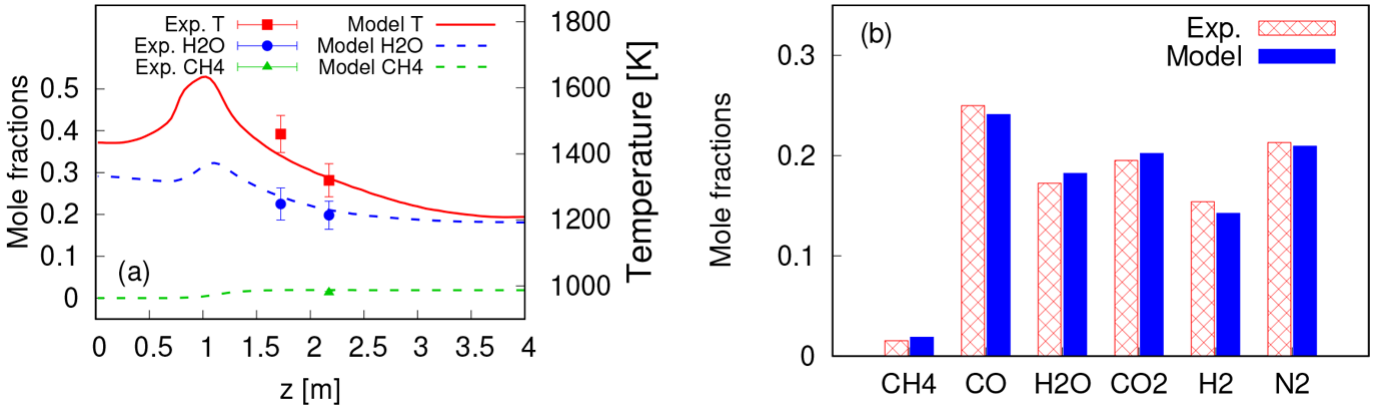
Validation of the numerical model. Average temperature, H_2_O, and CH_4_ concentrations along the reactor vertical axis compared to TDLAS measurements at port 1^35^ and port 2 (a), and mole fraction of major species at the outlet compared to FTIR and μGC measurements (b).

The numerical model is validated against the experimental measurements at the two optical ports and the gas mixture at the outlet. The temperature and H_2_O concentration at port 1 were reported earlier.^33^Figure 3a shows the average temperature, H_2_O and CH_4_ mole fractions along the vertical axis of the reactor, and a comparison with the TDLAS measurements at the optical port 1 and port 2 in the reactor. The mole fraction of species at the reactor outlet is also compared with the FTIR and μGC measurements (Figure 3b). The numerical predictions of the temperature and species concentrations in the EFG are in good agreement with the experiments, and the errors are comparable to those reported in earlier studies.^70^ The relative errors for temperature predictions at ports 1 and 2 are 4.32% and 0.72%, respectively. The prediction errors for species concentrations relative to total concentration at the two ports and at the outlet are all below 2%. Hence, the model is used in the following to study the main characteristics of biomass conversion inside the EFG.

**Figure 4:**
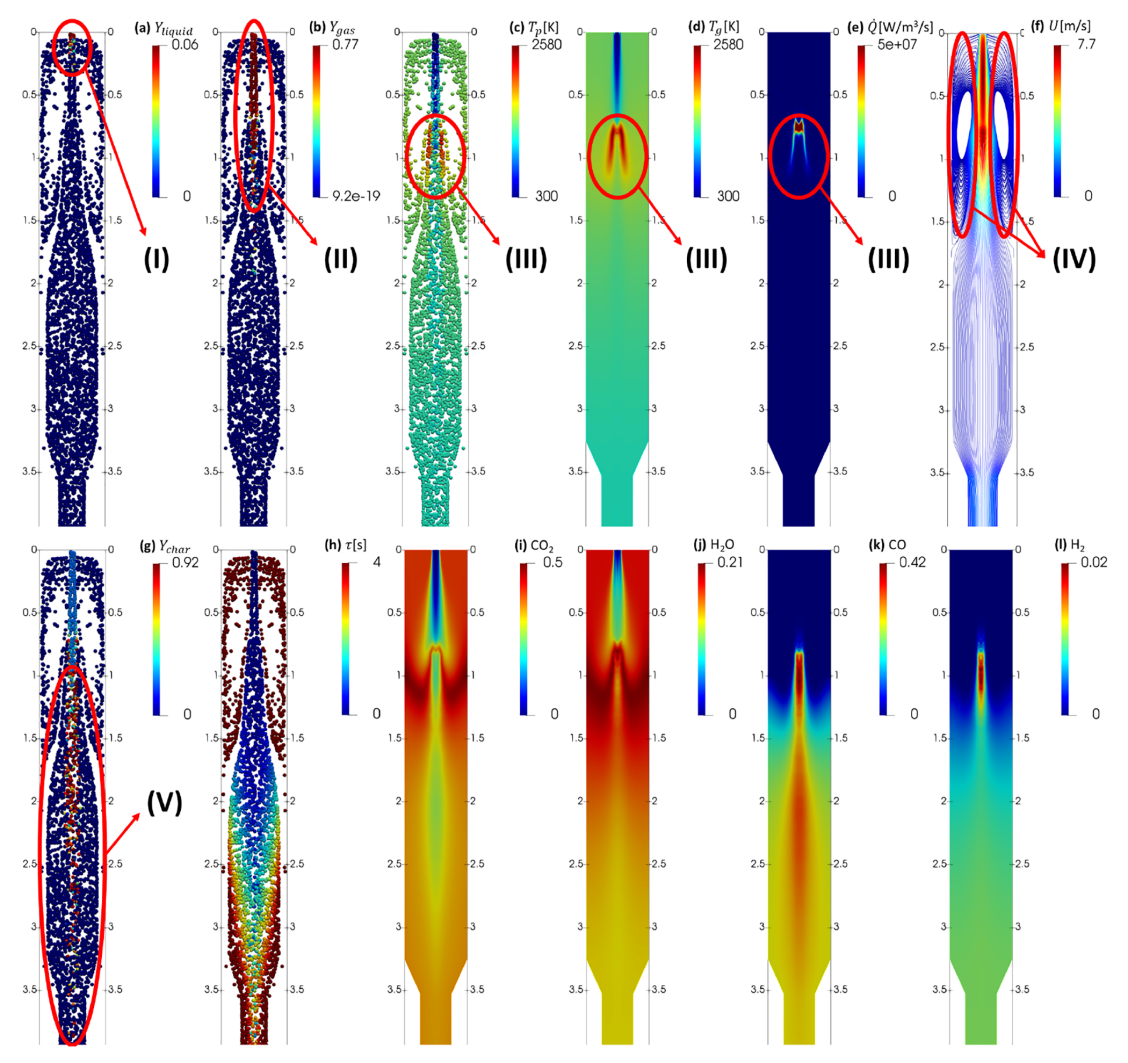
Numerical predictions for the solid parcels and gas-phase variables inside the EFG. The zones marked on the figures are (I) drying, (II) pyrolysis, (III) combustion, (IV) recirculation, and (V) gasification zones.

The gasification of biomass in an EFG is a complex process, and several different physiochemical processes are active at the same time in different zones inside the reactor. The location and size of different zones inside the gasifier, which are dependent on the operating conditions and the gasifier design, can be estimated using the simulation results. Each parcel of biomass particles undergoes drying, pyrolysis, and gasification or oxidation at different parts of the gasifier. Different quantities related to discrete particles and gas phase are used to explain the main processes in different zones in the reactor. Figure 4a shows the mass fraction of liquid water left in the biomass parcels and the drying zone, marked as zone I. The wet parcels are injected into the hot zone at the top of the reactor, and their moisture evaporates quickly in zone I. The volatile content of the parcels is presented in Figure 4b, where the pyrolysis zone is marked and labeled as zone II. Different zones may overlap because the size of the particles is different, and at a location where larger parcels are drying, the smaller parcels can undergo pyrolysis.

Zone III is the combustion zone and is marked in Figure 4c–e, which shows the parcels’ temperature, gas temperature, and heat generation due to gas-phase reactions, respectively. Both heterogeneous (R1) and homogeneous (R7–R10) reactions are active in this zone. The flame is stabilized by the recirculation zone IV off the center axis of the reactor as presented in Figure 4f along with the fluid flow streamlines. Since the reactor is operated in a fuel-rich condition, the combustion of solid char or gas-phase volatiles is incomplete. The partial combustion of the fuel is required in EFG to provide heat for the gasification process.

The mass fractions of char in the solid parcels and zone V are presented in Figure 4g. Zone V is the gasification zone and is the largest zone in the EFG. In this zone, the drying and pyrolysis are completed, and the solid parcels are mainly made of char and ash. The mass fraction of char decreases over time (moving from top to bottom) due to gasification. The mass fraction of char is higher in the middle of the EFG around the vertical axis. This can be attributed to the residence time of the parcels, τ, which is presented in Figure 4h. The parcels in the middle of the reactor have a higher momentum, and they reach the outlet faster than the surrounding parcels. Hence, the parcels in the middle have a shorter residence time which leads to a lower char conversion.

The main gasifying agents are CO_2_ and H_2_O, and the products of the gasification are CO and H_2_. The mass fractions of these four species are presented in Figure 4i–l. Part of H_2_O is formed during evaporation, and more H_2_O and CO_2_ are formed during pyrolysis and gas-phase combustion, which explains their maximum concentration close to the combustion zone III. In zone V, the concentration of CO_2_ and H_2_O decreases, and the concentration of CO and H_2_ increases due to gasification reactions. Similar trends are also observed in Figure 2b,c, where the average mole fraction of these species along the reactor axis is presented.

**Figure 5:**
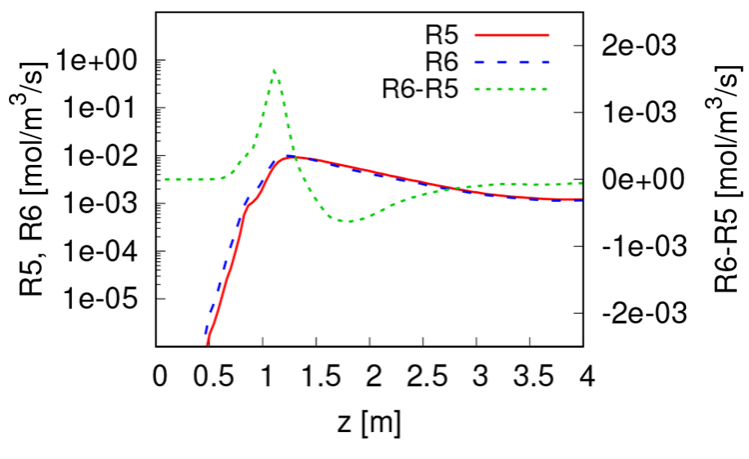
Rate of forward and backward water–gas shift reactions R5 and R6, and their difference.

The uncertainties on the kinetic rates for char gasification are very high. The reported values in the literature from various experiments show that the gasification with CO_2_ can be slower or faster than H_2_O (assuming the same partial pressure).^5^ Based on the kinetic rates that are considered in this work, and the conditions inside the EFG, the gasification with CO_2_ is orders of magnitude faster than gasification with H_2_O. Despite the very slow gasification with H_2_O, a considerable amount of H_2_O is consumed, and H_2_ is formed in the gasification region (Figure 2). This can be attributed to the water–gas shift reactions (R5 and R6) which are also active in the gasification region. The average rate of these reactions can be calculated by the average temperature and mole fractions of the species as presented in Figure 2. The rate of the forward and backward water–gas shift reactions is presented in Figure 5. The rates of the forward and backward water–gas shift reactions (R5 and R6) are too low at the top of the reactor where the concentrations of H_2_ and CO are low. The R5 and R6 rates become more significant around and downstream of the combustion zone. The rates of R5 and R6 have the same order of magnitude everywhere inside the reactor, which means that they are fast enough to almost reach the chemical equilibrium. Still, R6 is slightly faster in the gasification zone (from *z* = 1.2 to 3 m), which leads to H_2_ formation and H_2_O consumption as observed in Figure 2.

### 4.3 Potassium Release

**Figure 6:**
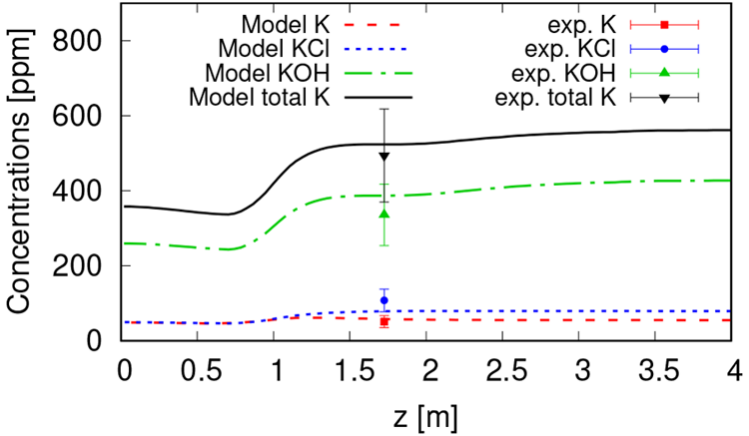
Concentration of different K species along the reactor vertical axis, compared to experimental measurements at port 1.^35^

The average concentration of main gas-phase K-containing species, i.e., K, KCl, and KOH, along the reactor vertical axis is presented in Figure 6 and compared with experimental measurements at port 1.^35^ The model predictions for all species and total K are within the range of experimental uncertainties. The prediction errors relative to the total K concentration are equal to 1.4%, 9.8%, 5.5%, and 5.65% for atomic K, KOH, KCl, and total K, respectively.

**Figure 7:**
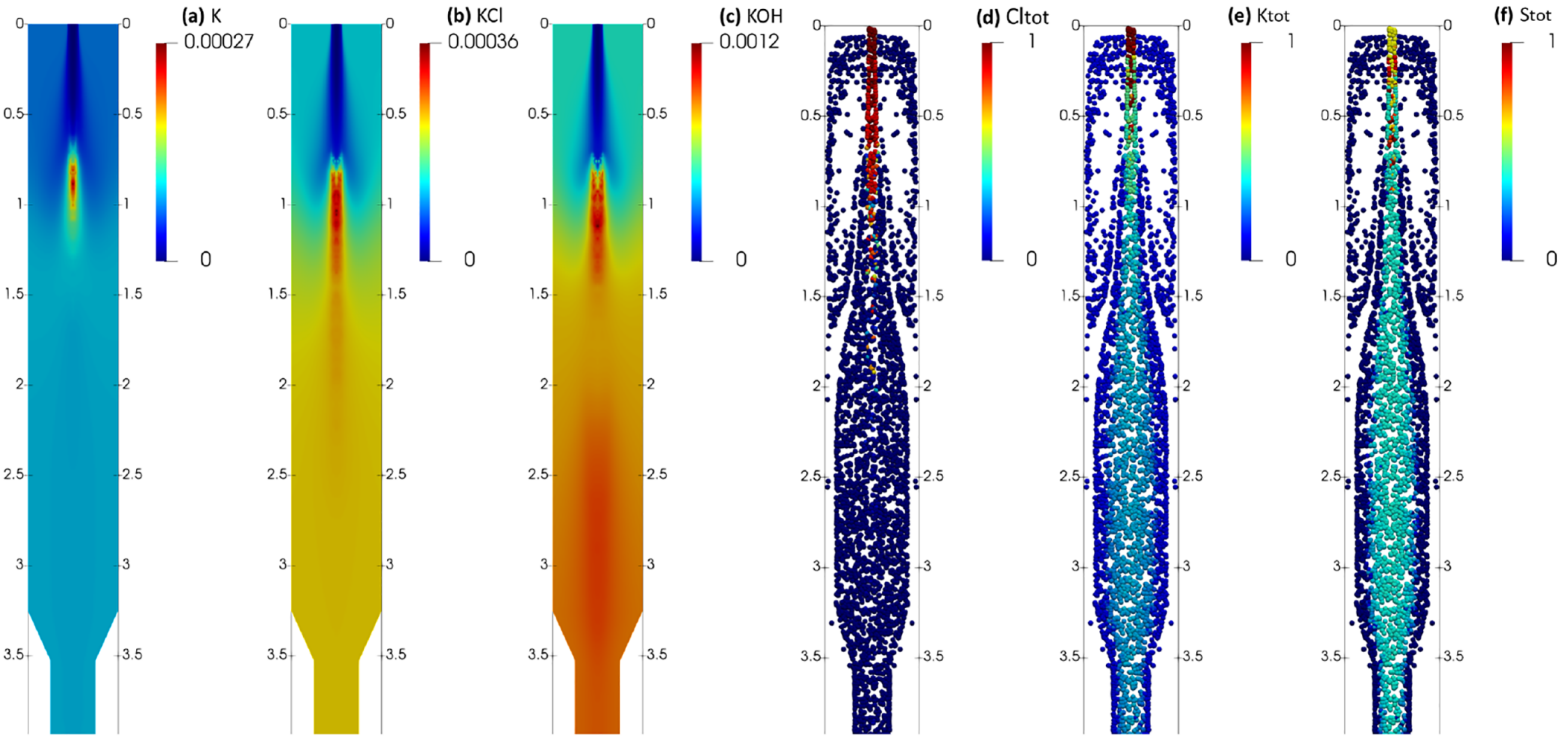
Mass fraction of K (a), KCl (b), and KOH (c) in the gas phase, and normalized mass of total Cl (d), total K (e), and total S (f) elements left in the solid particles.

The distribution of K, KCl, and KOH and also the residual mass of Cl, K, and S elements inside the gasifier are presented in Figure 7. The release of atomic K from organic K starts relatively early and is finished in the combustion zone, which explains its high concentration in the combustion zone (Figure 7a). KCl release starts at a higher temperature in the combustion zone (Figure 7b), and it continues until there is no more Cl left in the particles (Figure 7d). A major part of Cl is released as KCl in the combustion zone, and Cl release is almost finished 2 m downstream of the inlet. A significant amount of KOH is also released in the combustion zone (Figure 7c), but more KOH is released from the particles in the gasification zone. This can be explained by the considerable amounts of K (Figure 7e) and S (Figure 7f) that are still available in the solid particles in the gasification zone. In the gasification zone, a part of the residual K and almost all of the residual S in the solid are in the form of K_2_SO_4_, which slowly dissociates with water vapor (PR11) and forms more KOH. Based on Figure 7e,f, the particles that are closer to the reactor walls have a higher K and S release, which can be attributed to a longer residence time, or higher temperature and H_2_O concentration (Figure 4c,h,j) compared to the particles close to the center axis of the reactor.

**Figure 8:**
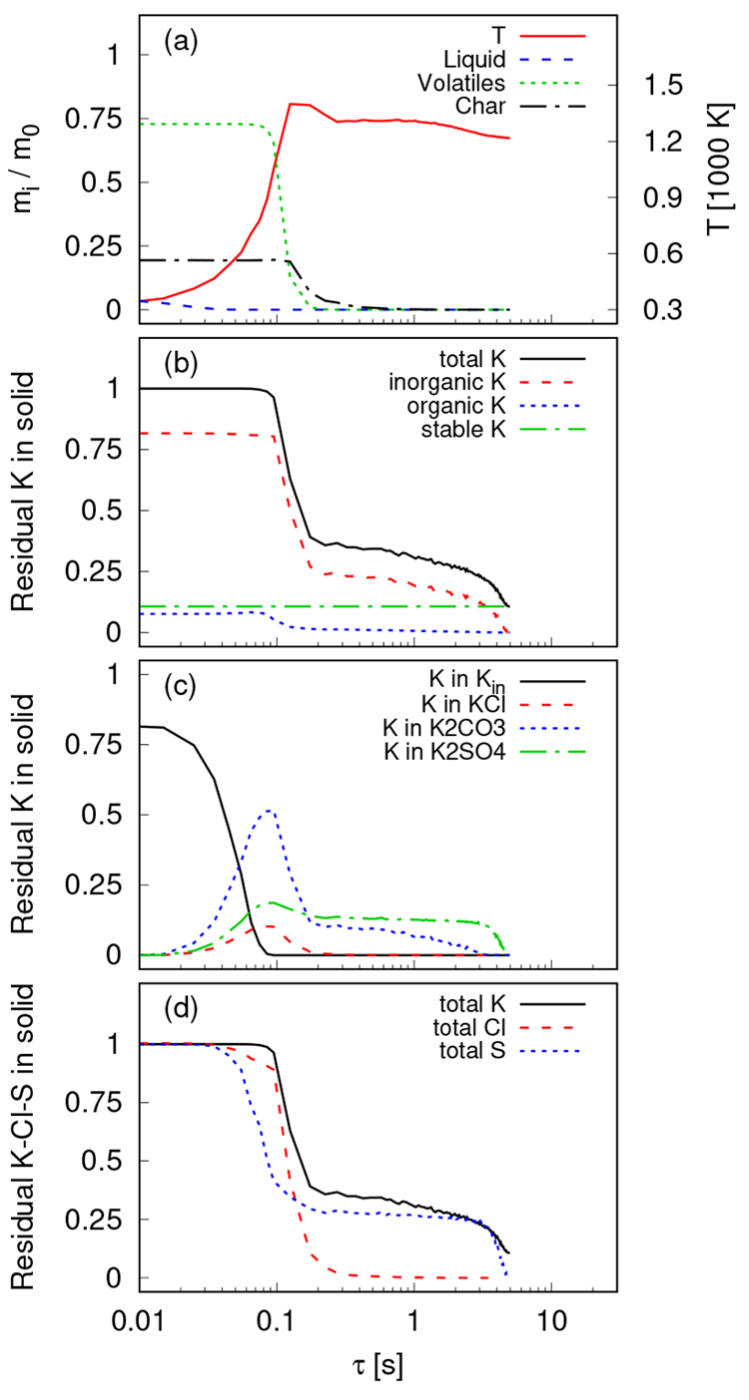
Averaged properties of particles as a function of residence time: parcel temperature, and normalized mass of liquid, gas, and solid (a); different types of residual K in solid (b); transformation of inorganic H_2_O-soluble K species (c); and normalized mass of K, Cl, and S elements in the solid residue (d).

The evolution of biomass particles and the solid-phase K species as a function of residence time, τ, is studied using the model, and the results are presented in Figure 8. There were more than 126,000 parcels in the domain. The parcels are divided into several bins based on their residence time between 0 and 5 s, and the ensemble average of the parcel properties in each bin is presented. Figure 8a shows the average temperature and the normalized mass of liquid, gas, and solid in the parcels. The particle drying is relatively fast, and almost all of the liquid water evaporates before 0.1 s. The temperature of the parcels increases more rapidly after the evaporation stage, and when the temperature is high enough around 0.1 s, the pyrolysis starts. At this stage, the normalized mass of gases inside the parcels (volatile content) decreases rapidly due to devolatilization. During pyrolysis between 0.1 and 0.2 s, the temperature of the parcels peaks due to the volatile combustion in the gas phase or partial combustion of char. Where there is no oxygen available, the endothermic gasification of char starts which leads to char consumption and a slow decrease in parcel temperature.

The potassium in the solid can be divided into three main groups which are inorganic (H_2_O-soluble), organic (NH_4_AC-soluble), and stable (acid or nonsoluble).^20^ In the present K-release model,^49^ the inorganic K species are initial K_inorganic_ and inorganic salts, i.e., KCl, K_2_CO_3_, and K_2_SO_4_. Initial K_organic_ and char-K are the organic species, and K_2_SiO_3_ (representative of all aluminosilicate-K species) is the stable K species which remains in the form of a solid until very high temperatures. The different types of K species and the total K in the solid are presented in Figure 8b. During the early stages of pyrolysis, organic K can be directly released to the gas phase in the form of atomic K, or it can be transformed to char-K which will be released during char conversion at higher temperatures. A higher temperature favors more gas-phase K-release compared to char-K formation.^49^ Hence, in this case, the majority of organic K is directly released to the gas phase around *t* = 100 ms in the form of atomic K. For Si-rich fuels, more K can be trapped in the aluminosilicate matrix during char conversion which leads to a higher fraction of stable K after conversion.^19,20,49^ However, in the case of Si-lean fuel such as FR in this study, Si is not sufficiently available for reaction with K, so the mass of stable K remains almost constant during conversion.

The majority of the potassium in the solid exists in H_2_O-soluble, inorganic form. The release of inorganic K starts at a higher temperature compared to the organic K. The transformation of inorganic K species is presented in Figure 8c. During drying, the inorganic K crystallizes and the inorganic salts are formed. The evaporation of KCl starts at a lower temperature (around 973 K) compared to the other two salts, and almost all of KCl is evaporated during the volatile combustion stage when the particle temperature is at its maximum. The rate of release of K from K_2_CO_3_ and K_2_SO_4_ depends on both temperature and water vapor concentration.^19,49^ Both the temperature and water concentration are maximum in the combustion zone (zone III in Figure 4), which explains the high release rate of K from K_2_CO_3_ and K_2_SO_4_ during 100–200 ms. This is in agreement with other experiments on a small batch of biomass particles where the maximum K-release was observed during the combustion stage.^21,32^ The evaporation and dissociation of K_2_CO_3_ and K_2_SO_4_ continue during the gasification stage, but the release of K from K_2_CO_3_ has a higher rate. The gasifier has a high water vapor concentration, so the dissociation rate of the salts will be orders of magnitude higher than their evaporation rate,^19^ which leads to a high amount of KOH formation.

The residual mass of Cl and S elements in the solid particle are presented as a function of residence time in Figure 8d together with the total K which was discussed earlier. The release of S happens in two stages. The first stage is the release of organic S^2–^ which is crystallized first and is then released as SO_2_ at low temperatures.^19,71^ The second stage of S release, which is relatively slow for Si-lean fuels,^19^ is related to the dissociation of K_2_SO_4_ with water vapor.

The release of Cl also happens in two consecutive stages. In the model, all Cl is initially considered to be present in the form of inorganic KCl salt.^49^ However, during the decomposition of hemicellulose, some amount of Cl will be released to the gas phase in the form of HCl, due to the reaction of KCl with carboxyl groups in the hemicellulose.^20,72^ This explains the first stage of the Cl-release that is happening before *t* = 100 ms. Right after the first stage, when the volatile combustion starts, the particle temperature rises, and the KCl evaporation becomes significant. Almost all particle Cl content is released after KCl evaporation, which is in agreement with the experimental observations in other studies.^19,22^

#### 4.3.1 Assessment of Model Assumptions

In the present work, the release of K species from solid particles is modeled, but the gas-phase reactions of K species after the release are neglected. Detailed^73^ and reduced^74^ mechanisms for the gas-phase reactions of K species are available in the literature. The detailed mechanisms in general are not suited for CFD simulations because of their high computational cost. Even a reduced mechanism cannot be used in this study, because not only does it significantly increase the computational cost, but it also requires the concentrations of O and H radicals which cannot be predicted by the global gas-phase reactions used in the present simulations. However, the error that is introduced to the model because of neglecting the gas-phase reactions of the K species is justified, as explained in the following.

**Figure 9:**
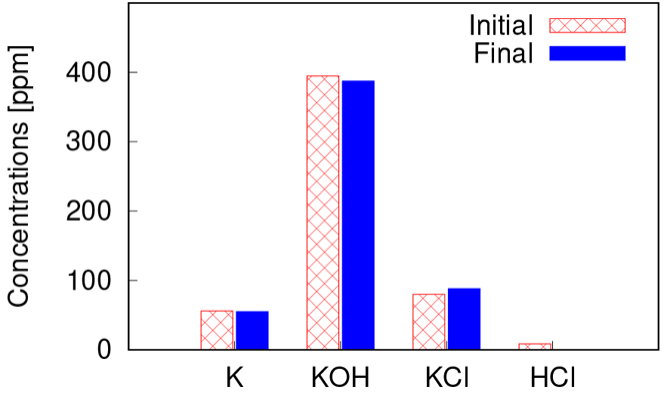
Initial and final concentration of K, KOH, KCl, and HCl, after 2 s reactions in a PSR initially under port 1 conditions.

The effects of the K species gas-phase reactions on the results are investigated using a 0D simulation of a perfectly stirred reactor (PSR). The reduced mechanism of Mortensen et al.^74^ was used for the simulation of the PSR with initial values of *T*, *p*, and concentrations similar to those at port 1 in the gasifier. The initial and final concentrations of K species and HCl after 2 s reactions in the PSR (to make sure equilibrium condition is reached) are presented in Figure 9. It was observed that the concentrations of K, KOH, and HCl decrease and that of KCl increases slightly due to the gas-phase reactions. In this case, almost all HCl is consumed in reaction with potassium from K and KOH which leads to KCl formation. However, the FR fuel in this study has a relatively low Cl content, and a large fraction of the Cl is released in the form of KCl. Hence, the concentration of HCl is very low in this case (9 ppm at port 1). Therefore, the effect of gas-phase reactions of K species is negligible in this case, and the error introduced for all K species is less than 10 ppm.

**Figure 10:**
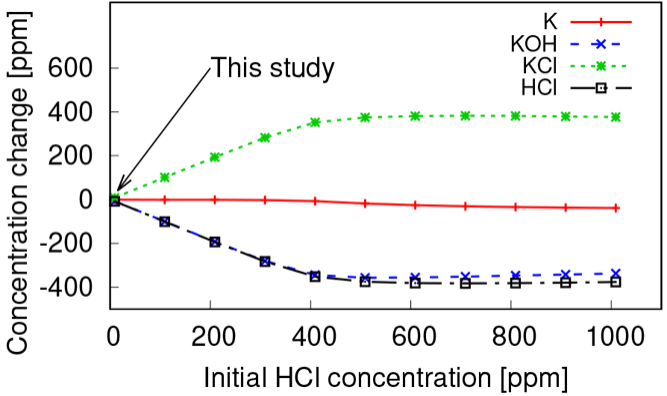
Effect of initial HCl concentration in the PSR on concentration change of K, KCl, KOH, and HCl after 2 s reactions.

In order to study the importance of the gas-phase reactions of K species, PSR simulations are carried out with 10 different initial concentrations of HCl. Only the HCl and N_2_ concentrations are varied compared to the earlier case corresponding to port 1 conditions. The change of concentration of K, KOH, KCl, and HCl after 2 s reactions (equilibrium) is presented in Figure 10. The point corresponding to port 1 in this study is also marked on the same figure. Based on the results, KCl is more stable than HCl in the gasification condition, so in every case, almost all of the HCl is reacted with KOH to form KCl. In cases with an initial HCl concentration of greater than 400 ppm, almost all KOH and K are consumed, and potassium is mainly available in KCl. It can be concluded that, in gasification conditions in the present study, Cl in the gas phase is expected to be available mainly in the form of KCl, and HCl is only expected for fuels with a high Cl/K molar ratio.

Another assumption used in the K-release model is the initial mass fractions of K, Cl, and S in different forms. The ratio of organic/inorganic elements in the FR fuel used in this study is unknown, so some values are adopted from the literature. Based on studies on different biomass sources, around 80% of K is in inorganic form, up to around 10% in a stable form, and the rest in organic form.^10,20,75^ On the other hand, between 40% and 60% of S is in organic form. Here, we have followed the measurements by Huang et al.^20^ and assumed that the fractions of inorganic, organic, and stable K are 81.8%, 7.5%, and 10.7% of total K in fuel, respectively. All Cl content was assumed to be in the form of inorganic KCl,^76^ and 60% of S was in organic form.^19^ In this section, the sensitivity of the model predictions to the origin of K–Cl–S elements is studied. A new set of PSR simulations are carried out, in which a single particle is gasified in similar conditions (temperature and H_2_O and CO_2_ mass fractions) to port 1. The size of the reactor is large compared to a single particle, so the temperature and mass fractions of H_2_O and CO_2_ remain constant during conversion. The sensitivities of the gas-phase K species to three parameters, i.e., the initial mass fraction of inorganic K, organic S, and stable K, are studied. Only one parameter is changed at a time, and PSR simulation is carried out for 2 s of reactions; the final molar ratio of released K, KOH, KCl, and total K to the initial K content of the original fuel is presented in Table 8.

**Table 8:** Change in the Gas-Phase K-Containing Products Due to Change in the Mass Fraction of Initial Inorganic K, Organic S, and Stable K^a^

variables	mole fraction of released K			
inorganic K	K	KOH	KCL	total K
0.70	0.16	0.30	0.11	0.56
0.75	0.12	0.32	0.11	0.55
*0.82*	*0.08*	*0.37*	*0.11*	*0.55*
0.85	0.05	0.38	0.11	0.54
0.89	0.02	0.40	0.11	0.53

organic S	K	KOH	KCL	total K
0.40	0.07	0.30	0.11	0.48
0.45	0.07	0.32	0.11	0.50
0.50	0.08	0.33	0.11	0.51
0.55	0.08	0.35	0.11	0.53
*0.60*	*0.08*	*0.37*	*0.11*	*0.55*

stable K	K	KOH	KCL	total K
0.05	0.10	0.39	0.11	0.59
0.08	0.09	0.38	0.11	0.57
*0.11*	*0.08*	*0.37*	*0.11*	*0.55*
0.13	0.07	0.36	0.11	0.53
0.15	0.06	0.35	0.11	0.51

a The italicized values correspond to the parameters used in EFG simulations.

According to the results, the KCl mole fraction is not sensitive to the mentioned parameters because its initial value is fixed, but the amount of K and KOH can change. A higher inorganic K (lower organic K) leads to a lower K and a higher KOH release. The release of atomic K is not sensitive to organic S, but a higher organic S leads to a higher KOH release. This is caused by the fact that a higher organic S is equivalent to a lower K_2_SO_4_ and a higher K_2_CO_3_ in the solid. The dissociation of K_2_CO_3_ with H_2_O is faster than that of K_2_SO_4_ which leads to a higher KOH formation. Finally, a higher stable K leads to a lower organic and inorganic K and, hence, a lower K, KOH, and total K. In all cases studied in this section, the total release of K species is affected by less than 8%, which is an acceptable value considering the complexity of the problem. The best way to reduce the uncertainties of the K-release model is to measure the origins of different elements in the fuel.

The numerical simulations in the present study were mainly aimed to study the performance and K-release from the EFG under specific operating conditions. The results can be used to understand the main phenomena that occur at different locations inside the reactor and the main parameters that are effective in the release of K in different forms. The same model can be used in future studies to investigate the effect of different operating parameters such as air–fuel ratio or fuel type on the performance, efficiency, and quality of produced biogas in EFGs. Furthermore, it was argued that the potassium release, which is dependent on many factors such as conversion atmosphere and ash composition of the fuel, can be predicted relatively well with the proposed K-release model. Hence, the model can be used to investigate different operating conditions to minimize the negative impact of inorganic elements on the EFG performance. To make the model more generally applicable, a detailed chemistry model for gasification should be developed, which also allows the modeling of the gas-phase potassium reactions. Further model validation can be attempted with detailed measurements in smaller experimental settings, such as single-particle reactors, with even better controlled operating conditions. Finally, only the release of K–Cl–S species was studied in this study, but to have a more comprehensive model in the future, the reactions of evolved K species with reactor surfaces should also be included in the model.

## 5 Conclusions

CFD simulation of a 140 kW entrained flow gasifier is carried out, where the model is validated against the experimental data using measurements from two optical access ports and also the gas composition at the outlet. Furthermore, a detailed K-release model developed and validated earlier is coupled to the CFD solver, and the transformation and release of K–Cl–S-containing species inside the reactor are studied. The concentrations of gas-phase K, KCl, and KOH at port 1 inside the gasifier are compared with the experimental measurements. The simulation results in this paper have led to the following conclusions:

- Five different zones are identified in the gasifier, where drying, pyrolysis, combustion, recirculation, and gasification reactions are the main processes in each zone, and the main characteristics of the zones are explained using the modeling results. The identified zones may overlap because of the differences in the size and conversion time of the biomass particles.
- It was found that the water–gas shift reaction is very fast in the gasification zone, so the forward and backward reactions are almost always in balance. Hence, the ratio between CO, H_2_O, CO_2_, and H_2_ is mainly determined by the water–gas shift reactions, rather than the gasification of char with H_2_O and CO_2_.
- The transformation and release of different types of K from forest residue in this gasifier are studied using the presented model. The model predictions of K, KCl, KOH, and total K had a relative error of 1.4%, 9.8%, 5.5%, and 5.7%, respectively, in comparison with PF-TDLAS measurements.
- A considerable amount of KOH is formed in the combustion zone, where the temperature and concentration of H_2_O are high. Therefore, the total K concentration around the combustion zone (from 0.7 to 1.3 m downstream of the inlet) rapidly increases from 336 to 510 ppm. The release of K in the form of KOH continues in the gasification zone, albeit at a lower rate. Hence, from 1.3 m to the outlet, the total K concentration increases from 510 to 561 ppm.
- It was shown that, for forest residue with a relatively low Cl content, neglecting the gas-phase reactions of potassium species has a minor effect (less than 10 ppm difference for all species) on the model predictions. Furthermore, it was found that K and KOH compared to KCl and total K predictions are more sensitive to the initial fraction of organic, inorganic, and stable K and S in the fuel. The prediction of total K-release showed less than 8% sensitivity to the changes in the initial fractions of K and S within the proposed range.
